# Supplementary material for: From sequence to enzyme mechanism using multi-label machine learning
Source: BMC Bioinformatics. 2014 May 19;15:150. doi: 10.1186/1471-2105-15-150 (PMC4229970; doi:10.1186/1471-2105-15-150)
Supplement: Additional file 2 — Java code of ml2db. Additional file ml2db_code.tar.gz contains the Java source code to run the multi-label machine learning experiments and save the results to database. The code’s Javadoc is included. [file 1471-2105-15-150-S2.zip › additional file 2/ml2db/ml2db/doc/mulan/classifier/transformation/CalibratedLabelRanking.html]

CalibratedLabelRanking


JavaScript is disabled on your browser.


- Overview
- Package
- Class
- Use
- Tree
- Deprecated
- Index
- Help

- Prev Class
- Next Class

- Frames
- No Frames

- All Classes

- Summary:
- Nested |
- Field |
- Constr |
- Method

- Detail:
- Field |
- Constr |
- Method


mulan.classifier.transformation

## Class CalibratedLabelRanking

- java.lang.Object
- - mulan.classifier.MultiLabelLearnerBase
  - - mulan.classifier.transformation.TransformationBasedMultiLabelLearner
    - - mulan.classifier.transformation.CalibratedLabelRanking

- All Implemented Interfaces:
  :   java.io.Serializable, MultiLabelLearner, weka.core.TechnicalInformationHandler

  ---

    

  ```
  public class CalibratedLabelRanking
  extends TransformationBasedMultiLabelLearner
  ```

  Class implementing the Calibrated Label Ranking (CLR) algorithm. For more information, see

  Version:
  :   2012.02.27

  Author:
  :   Elise Rairat, Sang-Hyeun Park, Grigorios Tsoumakas

  See Also:
  :   Serialized Form

- - ### Constructor Summary

    Constructors

    Constructor and Description

    `CalibratedLabelRanking()`

    Default constructor using J48 as underlying classifier

    `CalibratedLabelRanking(weka.classifiers.Classifier classifier)`

    Constructor that initializes the learner with a base algorithm
  - ### Method Summary

    Methods

    | Modifier and Type | Method and Description |
    | --- | --- |
    | `boolean` | `getStandardVoting()` Get whether standard voting is turned on. |
    | `weka.core.TechnicalInformation` | `getTechnicalInformation()` Returns an instance of a TechnicalInformation object, containing detailed information about the technical background of this class, e.g., paper reference or book this class is based on. |
    | `java.lang.String` | `globalInfo()` Returns a string describing the classifier. |
    | `MultiLabelOutput` | `makePredictionQW(weka.core.Instance instance)` This method does a prediction for an instance with the values of label missing according to QWeighted algorithm for Multilabel Classification (QCMLPP2), which is described in : Loza Mencia, E., Park, S.-H., and Fuernkranz, J. |
    | `MultiLabelOutput` | `makePredictionStandard(weka.core.Instance instance)` This method does a prediction for an instance with the values of label missing |
    | `void` | `setStandardVoting(boolean standardVoting)` Set Prediction to standard voting mode. |

    - ### Methods inherited from class mulan.classifier.transformation.TransformationBasedMultiLabelLearner

      `getBaseClassifier`
    - ### Methods inherited from class mulan.classifier.MultiLabelLearnerBase

      `build, getDebug, isUpdatable, makeCopy, makePrediction, setDebug`
    - ### Methods inherited from class java.lang.Object

      `equals, getClass, hashCode, notify, notifyAll, toString, wait, wait, wait`

- - ### Constructor Detail


    - #### CalibratedLabelRanking

      ```
      public CalibratedLabelRanking()
      ```

      Default constructor using J48 as underlying classifier


    - #### CalibratedLabelRanking

      ```
      public CalibratedLabelRanking(weka.classifiers.Classifier classifier)
      ```

      Constructor that initializes the learner with a base algorithm

      Parameters:
      :   `classifier` - the binary classification algorithm to use
  - ### Method Detail


    - #### setStandardVoting

      ```
      public void setStandardVoting(boolean standardVoting)
      ```

      Set Prediction to standard voting mode.

      Parameters:
      :   `standardVoting` - `true` if standard voting should be used


    - #### getStandardVoting

      ```
      public boolean getStandardVoting()
      ```

      Get whether standard voting is turned on.

      Returns:
      :   `true` if standard voting is on


    - #### makePredictionStandard

      ```
      public MultiLabelOutput makePredictionStandard(weka.core.Instance instance)
                                              throws java.lang.Exception
      ```

      This method does a prediction for an instance with the values of label missing

      Parameters:
      :   `instance` -

      Returns:
      :   prediction

      Throws:
      :   `java.lang.Exception`


    - #### makePredictionQW

      ```
      public MultiLabelOutput makePredictionQW(weka.core.Instance instance)
                                        throws java.lang.Exception
      ```

      This method does a prediction for an instance with the values of label missing
      according to QWeighted algorithm for Multilabel Classification (QCMLPP2), which is
      described in :
      Loza Mencia, E., Park, S.-H., and Fuernkranz, J. (2009)
      Efficient voting prediction for pairwise multilabel classification.
      In Proceedings of 17th European Symposium on Artificial
      Neural Networks (ESANN 2009), Bruges (Belgium), April 2009
      This method reduces the number of classifier evaluations and guarantees the same
      Multilabel Output as ordinary Voting. But: the estimated confidences are only
      approximated. Therefore, ranking-based performances are worse than ordinary voting.

      Parameters:
      :   `instance` -

      Returns:
      :   prediction

      Throws:
      :   `java.lang.Exception`


    - #### globalInfo

      ```
      public java.lang.String globalInfo()
      ```

      Returns a string describing the classifier.

      **Overrides:**
      :   `globalInfo` in class `TransformationBasedMultiLabelLearner`

      Returns:
      :   a string description of the classifier


    - #### getTechnicalInformation

      ```
      public weka.core.TechnicalInformation getTechnicalInformation()
      ```

      **Description copied from class: `TransformationBasedMultiLabelLearner`**

      Returns an instance of a TechnicalInformation object, containing detailed
      information about the technical background of this class, e.g., paper
      reference or book this class is based on.

      **Specified by:**
      :   `getTechnicalInformation` in interface `weka.core.TechnicalInformationHandler`

      **Overrides:**
      :   `getTechnicalInformation` in class `TransformationBasedMultiLabelLearner`

      Returns:
      :   the technical information about this class


- Overview
- Package
- Class
- Use
- Tree
- Deprecated
- Index
- Help

- Prev Class
- Next Class

- Frames
- No Frames

- All Classes

- Summary:
- Nested |
- Field |
- Constr |
- Method

- Detail:
- Field |
- Constr |
- Method
